# Variability on particulate matter and meteorology dataset during the hazy period in eastern region of Peninsular Malaysia

**DOI:** 10.1016/j.dib.2020.105210

**Published:** 2020-02-01

**Authors:** Ku Mohd Kalkausar Ku Yusof, Siti Sofo Ismail, Azman Azid, Muhamad Shirwan Abdullah Sani, Noorain Mohd Isa, Mohd Zulfahmie Mohamat Zawawi

**Affiliations:** a Faculty of Science and Marine Environment, Universiti Malaysia Terengganu, 21300, Kuala Terengganu, Terengganu, Malaysia; b Faculty of Bioresources and Food Industry, Universiti Sultan Zainal Abidin (UniSZA), Besut Campus, 22200 Besut, Terengganu, Malaysia; c International Institute for Halal Research and Training, Level 3, KICT Building, International Islamic University Malaysia, Jalan Gombak, 53100, Kuala Lumpur, Malaysia; d Faculty of Environmental Studies, Universiti Putra Malaysia (UPM), 43400 Serdang, Selangor, Malaysia; e NHS Environmental Services Sdn. Bhd. No.7, Jalan 3/25, Seksyen 3, 43650 Bandar Baru Bangi, Selangor, Malaysia

**Keywords:** Haze, Histogram, Normality test, Meteorological, Particulate matter (PM_10_)

## Abstract

This paper provides detail on sequence analysis of hazy days based on eight monitoring stations from three states (Kelantan, Terengganu and Pahang) in the eastern region of Peninsular Malaysia. The dataset comprises of 1502 daily mean hazy days that had been measured for a decade. The meteorology data namely wind speed, wind direction, air temperature, relative humidity and particulate matter (PM_10_) were used to comprehend the variability, and the relationship existed amongst variables. The final dataset consists of a summary descriptive analysis and a boxplot, where all five variables were involved, including the minimum, maximum, mean, 1st quartile, median, 3rd quartile and standard deviation are presented. Apart from descriptive analysis, the normality test and histogram were performed as well.

**Table:** Specifications Table

Subject	Environmental Science
Specific subject area	Air Quality, Haze
Type of data	Table Figure
How data were acquired	The data was obtained from the Air Quality Division, Department of Environment, Malaysia (DOE). The types of equipment used to measure the air pollutant and meteorological data by DOE were summarised below; BAM-1020 Beta Attenuation Mass Monitor, Met One 010C, Met One 020C, Met One 062, Met One 083D,
Data format	Raw, Analysed
Parameters for data collection	Wind speed, wind direction, air temperature, relative humidity and particulate matter
Description of data collection	The raw dataset was obtained from the DOE. The data started in early January 2006 until the end of December 2015. The data then has been pre-treated before proceeding with further analysis. A minimum of 150 μg/m^3^ daily means for particulate matter (PM_10_) as per guideline by National Ambient Air Quality Standard (IT-1) (NAAQS) be used as a reference to identify the hazy day. Any daily mean of the meteorological dataset that concurrently measured was also selected.
Data source location	Kota Bharu, Kelantan, Malaysia (N06° 09.520, E102° 17.262) Kota Bharu, Kelantan, Malaysia (N06° 09.520, E102° 15.059) Kuala Terengganu, Terengganu, Malaysia (N05° 18.455, E°103 07.213) Kertih, Terengganu, Malaysia (N04° 35.880, E103° 26.096) Kemaman, Terengganu, Malaysia (N04° 16.260, E103° 25.826) Kuantan, Pahang, Malaysia (N03° 49.138, E103° 17.817) Kuantan, Pahang, Malaysia (N03° 57.726, E103° 22.955) Jerantut, Pahang, Malaysia (N03° 58.238, E103° 20.863)
Data accessibility	Public repository Repository: Mendeley Data https://doi.org/10.17632/5zjwxkd2zn.2 DOI: 10.17632/5zjwxkd2zn.2

**Table:** 

**Value of the Data** • The datasets incorporate a large number (a decade) of hazy days in the eastern region of Peninsular Malaysia along with a variety of meteorology data. • This dataset provides insights, which it can be used by researchers to understand the meteorology in Malaysia especially in eastern region datasets towards PM_10_ during haze period over a decade dataset. • The dataset discloses the variability of the meteorology variable during the hazy period.

## 1 Data description

Table 1 shows the normality test result from four different techniques, namely Shapiro-Wilk, Anderson-Darling, Lilliefors and Jarque-Berra. The normality test was conducted from five variables within a decade. The result demonstrated that the dataset of particulate matter (PM_10_), wind speed (WS), wind direction (WD), air temperature (AT) and relative humidity (RH) are not normal. Table 2 and Fig. 2 show the variability of all variables, i.e. the minimum, maximum, 1st quartile, median, 3rd quartile, mean and standard deviation. Fig. 2, Fig. 3 show the normality test and histogram for each variable, respectively.

**Table 1:** Summary of the normality test.

Variable∖Test	Shapiro-Wilk	Anderson-Darling	Lilliefors	Jarque-Bera
PM_10_	< 0.0001	< 0.0001	< 0.0001	< 0.0001
WS	< 0.0001	< 0.0001	< 0.0001	< 0.0001
WD	< 0.0001	< 0.0001	< 0.0001	< 0.0001
AT	< 0.0001	< 0.0001	< 0.0001	< 0.0001
RH	< 0.0001	< 0.0001	< 0.0001	< 0.0001

Values in bold are different from 0 with a significance level alpha = 0.95 (p-value: < 0.05).

**Table 2:** Descriptive analysis of particulate matter and meteorology dataset.

Statistic	Particulate Matter (PM_10_)			Wind speed			Wind direction			Temperature			Relative Humidity		
	KTN	TGU	PHG	KTN	TGU	PHG	KTN	TGU	PHG	KTN	TGU	PHG	KTN	TGU	PHG
Minimum	150.00	150.00	150.00	0.80	0.90	0.90	0.00	3.00	0.00	21.10	20.60	20.00	48.00	49.00	42.00
Maximum	364.00	476.73	435.00	14.60	14.50	23.20	358.00	359.00	360.00	34.50	38.20	34.80	100.00	100.00	101.00
1st Quartile	157.00	161.00	161.00	2.13	1.70	3.50	75.00	176.75	132.27	25.35	25.70	24.63	77.00	75.50	80.00
Median	168.00	174.63	177.58	3.55	2.92	4.90	172.08	218.00	228.15	27.00	27.10	26.10	82.50	83.55	87.00
3rd Quartile	186.00	204.38	204.25	5.10	5.49	7.20	221.75	264.75	305.19	28.60	29.40	28.20	88.00	91.97	93.00
Mean	178.01	193.38	190.28	3.88	3.91	5.74	161.43	214.01	208.68	26.99	27.53	26.48	81.18	83.30	85.73
Standard deviation	33.83	50.99	42.97	2.46	2.89	3.33	89.83	71.69	110.42	2.37	2.72	2.62	8.83	11.16	10.36

**Fig. 1:**
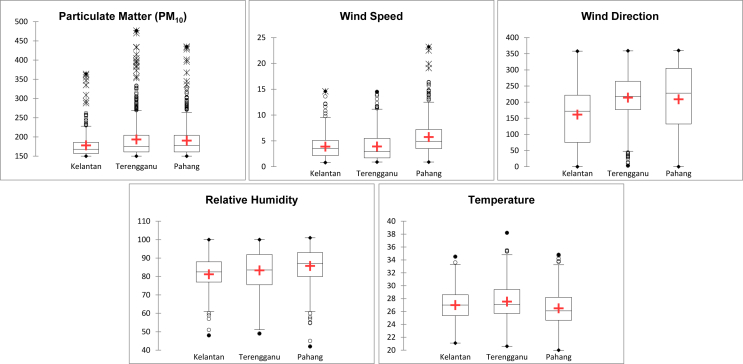
A decadal analysis (box plot) based on the hazy period for particulate matter and meteorology dataset.

**Fig. 2:**
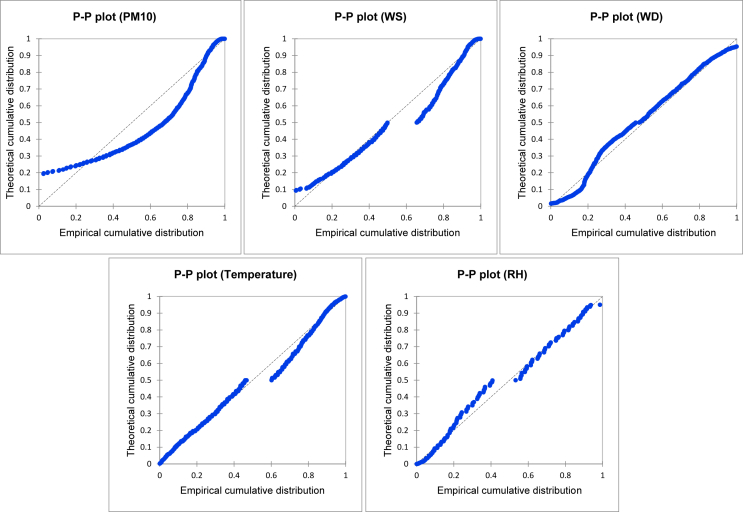
The normality test chart.

**Fig. 3:**
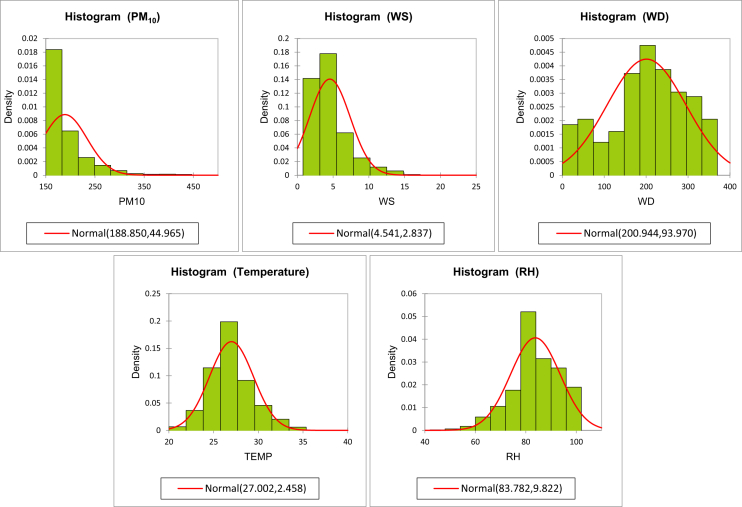
Histogram analysis of particulate matter and meteorology dataset.

From Fig. 1, the mean value for each variable is mostly equivalent in Kelantan (KTN), Terengganu (TGU) and Pahang (PHG). KTN was the only state that has low value for all types of variables. However, PHG consistently showed the differentiation in presenting the outliers in PM_10_, WS and RH.

## 2 Experimental design, materials, and methods

**Fig. 4:**
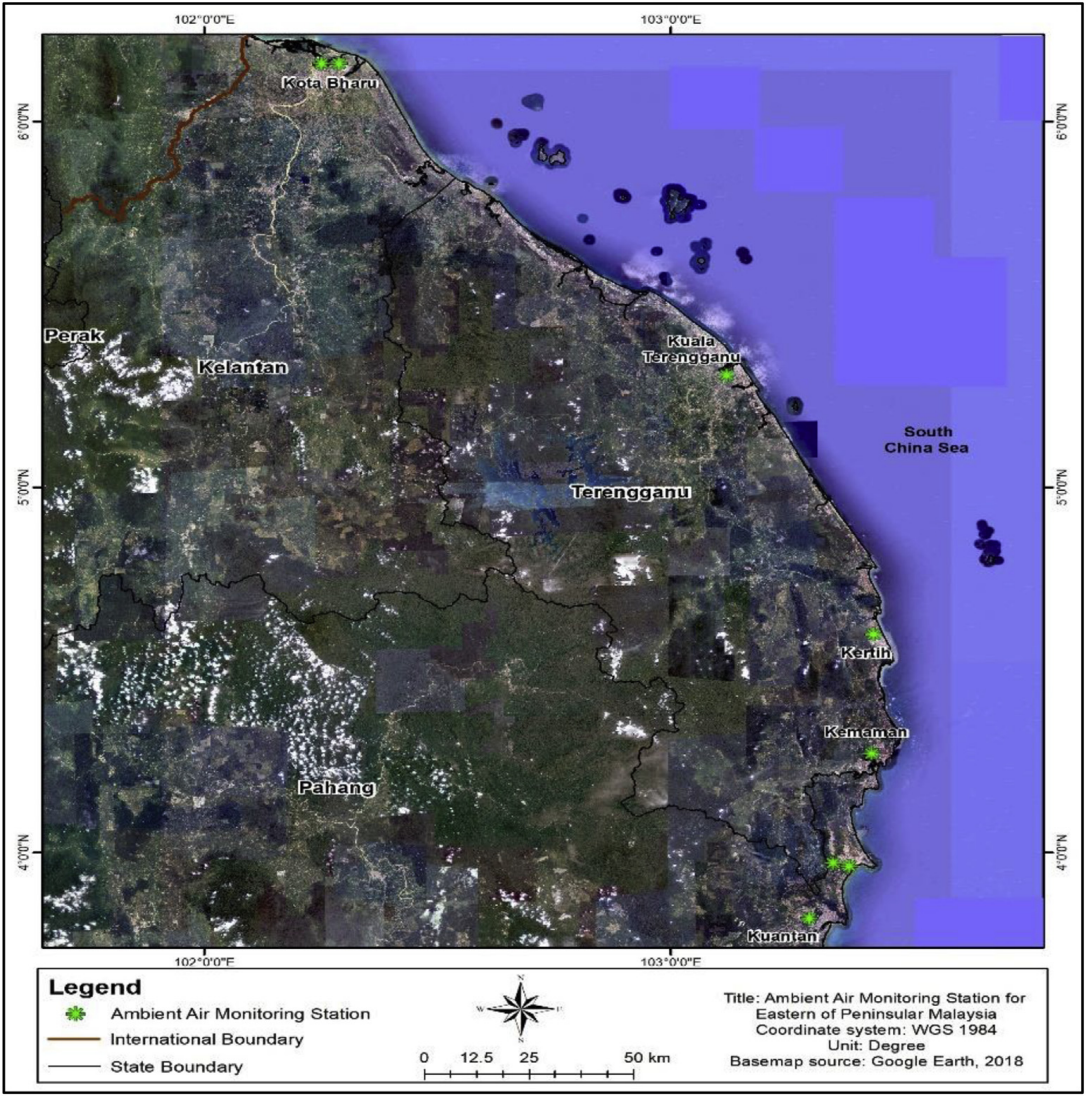
The Continuous Air Quality Monitoring (CAQM) station.

Figure 4 shows the locations for the Continuous Air Quality Monitoring (CAQM) at three different states. The dataset was obtained from the Department of Environment (DOE), Ministry of Science, Technology, Environment and Climate Change (MESTECC), Malaysia. All monitoring stations were managed and maintained by Alam Sekitar Malaysia Sdn. Bhd. (ASMA), a private company that officially hired by the Department of Environment (DOE) Malaysia. A total of 1502 daily observations, involving the five parameters, were identified as hazy. The dataset was selected from a daily mean series from January 2006 to December 2015. Eight monitoring stations in the eastern region of Peninsular Malaysia were involved, i.e. two stations in KTN, three stations in TGU and three stations in PHG. From eight monitoring stations, three stations were categorised with an industrial background. These stations are located in Kota Bharu (KTN), Kertih (TGU) and Kuantan (PHG). The other five stations were installed to monitor the air quality around the residential area.

The main criterion for hazy selection was based on two factors. Firstly, the dataset must be greater than 150 μg/m^3^ and categorised under unhealthy (API >101) status in the API level. Under the 1st interim of National Ambient Air Quality Standard (NAAQS), any measured particulate matter must be not exceeded more than 150 μg/m^3^ for the 24hours duration. Particulate matter was chosen compared to other API pollutant (SO_2_, NO_2_, O_3_, CO) parameters due to its unique characteristics during haze. It was proven that particulate matter is highly related to haze event [1,2].
